# Association of Single-Nucleotide Polymorphisms in Capecitabine Bioactivation Pathway with Adjuvant Therapy Safety in Colorectal Cancer Patients

**DOI:** 10.3390/pharmaceutics15112548

**Published:** 2023-10-28

**Authors:** Yasmin Cura, Almudena Sánchez-Martín, Noelia Márquez-Pete, Encarnación González-Flores, Fernando Martínez-Martínez, Cristina Pérez-Ramírez, Alberto Jiménez-Morales

**Affiliations:** 1 Pharmacy Service, Pharmacogenetics Unit, Hospital Universitario Virgen de las Nieves, 18014 Granada, Spain; 2 Medical Oncology, Hospital Universitario Virgen de las Nieves, 18014 Granada, Spain; 3 Biosanitary Research Institute, Ibs.Granada, 18012 Granada, Spain; 4 Pharmaceutical Care Research Group, Pharmacy Faculty, University of Granada, 18016 Granada, Spain; 5 Department of Biochemistry and Molecular Biology II, Institute of Nutrition and Food Technology “José Mataix”, Center of Biomedical Research, University of Granada, 18016 Granada, Spain

**Keywords:** capecitabine, pharmacogenetics, single-nucleotide polymorphisms, safety, toxicity, treatment suspension, colorectal cancer

## Abstract

Capecitabine, an oral prodrug of 5-fluorouracil (5-FU), is part of the standard treatment of colorectal cancer (CRC). Severe adverse dose limiting reactions that impair treatment safety and lead to treatment suspension remain a relevant concern. Single-nucleotide polymorphisms (SNPs) in genes involved in the activation of capecitabine may alter the bioavailability of 5-FU and thereby affect therapy outcomes. The aim of this study was to evaluate the association of these SNPs with severe toxicity and treatment suspension in patients with CRC treated with capecitabine-based therapy. An ambispective cohort study was conducted, including 161 patients with CRC. SNPs were analyzed using real-time PCR with TaqMan^®^ probes. Toxicity was assessed according to the National Cancer Institute Common Terminology Criteria for Adverse Events v.5.0. CES1 rs71647871-A was associated with a severe hand–foot syndrome (*p* = 0.030; OR = 11.92; 95% CI = 1.46–73.47; GG vs. A). CDA rs1048977-CC (*p* = 0.030; OR = 2.30; 95% CI 1.09–5.00; T vs. CC) and capecitabine monotherapy (*p* = 0.003; OR = 3.13; 95% CI 1.49–6.81) were associated with treatment suspension due to toxicity. SNPs CES1 rs71647871 and CDA rs1048977 may act as potential predictive biomarkers of safety in patients with CRC under capecitabine-based adjuvant therapy.

## 1. Introduction

Colorectal cancer (CRC) is a major health burden. The estimated annual incidence of CRC in 2023 is approximately 153,020 new cases in the U.S, representing approximately 8% of the total cancer incidence [1]. Adjuvant chemotherapy plays a crucial role in improving patient outcomes following surgical resection [2,3]. Capecitabine, an oral prodrug of fluoropyrimidine (FP) 5-fluorouracil (5-FU), is a widely used treatment option in the adjuvant setting for CRC. It offers comparable efficacy to intravenous 5-FU-based regimens while presenting a better safety profile and a greater convenience for patients [4,5]. 

**Figure 1:**
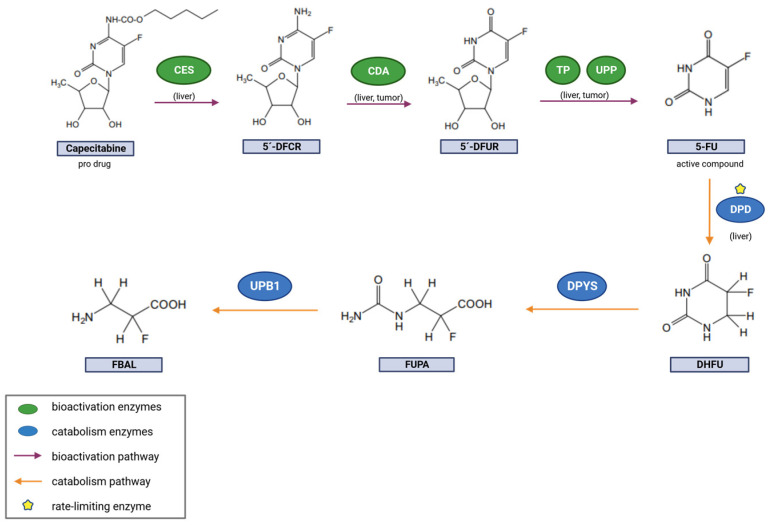
Capecitabine bioactivation and catabolism pathway. Created with BioRender.com.

Despite the effectiveness of capecitabine in treating CRC, it is not exempt from dose-limiting adverse reactions, which can lead to treatment suspension. The most frequent reported dose-limiting adverse reactions include the hand–foot syndrome (HFS), diarrhea, nausea, abdominal pain, and stomatitis [6]. Furthermore, considerable inter-patient variability exists regarding treatment response and toxicity in patients with CRC receiving capecitabine therapy. This variability in treatment outcomes has prompted investigations into potentially underlying genetic factors [7]. One of the most extensively studied genes in this context is dihydropyrimidine dehydrogenase (DPYD), which encodes the dihydropyrimidine dehydrogenase (DPD) enzyme primarily responsible for the catabolism of capecitabine [8,9,10,11]. Several single-nucleotide polymorphisms (SNPs) in this gene have been associated with an increased susceptibility to capecitabine treatment toxicity and, currently, clinical pharmacogenetic guidelines and medicine agencies recommend genotyping for four specific variants (rs3918290 (DPYD*2A), rs55886062 (DPYD*13), rs67376798, and rs56038477 (HapB3)) [12,13]. However, the genotyping of these SNPs alone has been found to only account for 30% of severe toxicity events associated with FP-based therapies [14]. This suggests that the remaining toxicity may be attributed to other genetic variants involved in the pharmacokinetics (PK) of capecitabine [15]. One pathway of particular interest in this regard is capecitabine’s bioactivation pathway. The activation of capecitabine involves a series of enzymatic reactions that ultimately transform the prodrug into its active metabolites. The initial enzymatic transformation of capecitabine takes place in the liver, primarily through the catalytic activity of the carboxylesterase 1 (CES1) enzyme. This enzymatic activity leads to the formation of 5′-deoxy-5-fluorocytidine (5′-dFCR). Following this initial step, the metabolite 5′-dFCR undergoes further metabolism by cytidine deaminase (CDA), resulting in the production of 5′-deoxy-5-fluorouridine (5′-dFUR). The final conversion to the active compound 5-FU takes place through the enzymatic action of either thymidine phosphorylase (TP) or uridine phosphorylase (UPP) (Figure 1). While TP, encoded by the thymidine phosphorylase gene (TYMP), is expressed in both liver and tumor tissues, and its expression is notably higher in tumor tissues [16,17]. Given the crucial role of these enzymes in the bioactivation pathway of capecitabine, genetic variants affecting their activity may have the potential to modify the level of conversion into 5-FU. Consequently, this may alter the bioavailability of the active drug and thereby influence therapeutic outcomes of capecitabine-based treatments.

Several SNPs in genes encoding capecitabine’s bioactivation enzymes have been investigated as potential determinants of treatment toxicity. However, the current body of evidence is contradictory, highlighting the need for further research in this area [18,19,20,21,22,23,24,25,26,27,28,29].

In this study, we investigated SNPs in the genes CES1/2, CES1P1, CDA, and TYMP, which encode enzymes involved in the bioactivation of capecitabine and could potentially influence susceptibility to treatment-related toxicity in patients with CRC.

## 2. Materials and Methods

### 2.1. Study Design and Population

This study was designed as an observational, ambispective, cohort study. Samples containing 50 µL DNA from patients with CRC who had their DPYD genotype determined at the Hospital Universitario Virgen de las Nieves, Granada, Spain, were requested to the Andalusian Public Health System biobank and stored at −40 °C. The inclusion criteria were diagnosis of CRC treated with adjuvant capecitabine-containing regimen, age ≥ 18 years, and performance status (PS), ≤2. Exclusion criteria were abnormal hematological, renal, or liver function, previous malignancies, recent or concomitant treatment with brivudine, and unavailable medical records. The number of patients with CRC that met selection criteria and had available DNA samples during the study recruitment period (2020–2022) determined the sample size. All patients were treated with capecitabine in monotherapy or combined with other antineoplastic strategies (oxaliplatin, bevacizumab, radiotherapy) for 14 days in 3-week cycles. Follow-up visits and laboratory analyses were performed at every treatment cycle completion and documented in medical records. Capecitabine-based therapy was administered until scheduled cycles were successfully completed, occurrence of unacceptable toxicity, disease progression, or death.

### 2.2. Ethical Considerations 

This study adhered to the ethical principles outlined in the Declaration of Helsinki. Prior approval was obtained from Biomedical Research Ethics Committee of Granada (identification code 0632-M2-20, 2020). Informed consent for donation to the Biobank of the Andalusian Public Health System was obtained from the participants, and strict confidentiality measures were implemented to protect their privacy.

### 2.3. Sociodemographic and Clinical Variables 

Sociodemographic and clinical variables that were collected included age, alcoholic and smoking habits, sex, tumor location, histology and size, cancer stage and grade, performance status, type of capecitabine-based adjuvant regimen, and treatment line. Data were extracted from medical records.

### 2.4. Safety Outcome Measures

The primary outcome of this study was capecitabine-based toxicity, defined as overall toxicity (any kind) or dose-limiting toxicity (diarrhea, abdominal pain, HFS, stomatitis, and nausea). Toxicity was graded according to the Common Terminology Criteria for Adverse Events v.5.0 (CTCAE) of the National Cancer Institute and further categorized into severe (grade ≥ 3) and mild (grade < 3) toxicity [30]. The secondary outcome was capecitabine-based treatment suspension due to any kind of severe toxicity categorized as yes/no. Toxicity and treatment suspension data were obtained from medical and laboratory test records.

### 2.5. Genetic Variables

The quality and quantity of DNA samples were measured using a spectrophotometer (NanoDrop 2000 UV). A total of 10 SNPs in genes CES1 (rs2244613 and rs71647871), CES1P1 (rs7187684 and rs11861118), CES2 (rs11075646), CDA (rs532545, rs602950, rs2072671, rs1048977), and TYMP (rs11479) were chosen based on the existing literature (Table S1) [18,19,20,21,22,23,24,25,26,27,28,29]. A variable representing the DPYD gene was included due to its potential as a confounding factor. DPYD status of the 4 clinically relevant variants (rs3918290, rs55886062, rs67376798, and rs56038477) was reviewed in medical records and categorized into DPYD variant carrier or non-carrier. Genotyping was performed in the pharmacogenetics unit of the hospital’s pharmacy service using the polymerase chain reaction (PCR) method with TaqMan probes on the QuantStudio^®^ 3 Real Time PCR System (96 wells; Thermo Fisher Scientific, Waltham, MA, USA), following the instructions provided by the manufacturer. The process was carried out in multiple batches to accommodate the sample size. To ensure genotyping results accuracy, a subset of 10% of the samples was randomly selected and analyzed in duplicate. The analysis revealed a perfect concordance between duplicate and original samples. Genotyping yielded successful results for all the selected SNPs included in the analysis, with call rates exceeding 98%. 

### 2.6. Statistical Methods

Quantitative data were summarized using median and interquartile range [p25–p75], while qualitative data were presented as frequencies and percentages. Normality of the data distribution was assessed using the Kolmogorov–Smirnov test. The genotype frequencies for all selected SNPs were examined for adherence to Hardy–Weinberg equilibrium (HWE). Software tools PLINK v1.9 and Haploview v.4.1 were utilized for linkage disequilibrium (LD) analysis and display [31,32]. Haplotype frequency inference and association analysis was performed in SNPstats tool [33]. Bivariate analysis was performed using Pearson’s chi-squared test or Fisher’s exact test to examine the association between safety outcome variables and the independent sociodemographic, clinical, and SNP variables (in genotypic, dominant, and recessive models). When possible, multivariate analysis was conducted using logistic regression (backward stepwise method) to explore the relationship between multiple independent variables (with *p*-value ≤ 0.05 in bivariate analysis) and safety outcomes. Adjusted odds ratios (ORs) and their corresponding 95% confidence intervals (95% CIs) were calculated to estimate association strength and direction. A *p*-value < 0.05 was considered statistically significant. False discovery rate (FDR) correction was applied to account for multiple comparisons in multivariate analysis. PLINK v1.9 and R v.4.2.2 (R Foundation for Statistical Computing, Vienna, Austria) software [31,34] were employed for data analysis.

## 3. Results

### 3.1. Sociodemographic and Clinical Characteristics

A total of 161 patients with CRC were included in this study. Baseline characteristics of the study population are presented in Table 1. Of all patients, 38.51% (62/161) were female, 40.99% (66/161) had rectal cancer, 78.88% had adenocarcinoma (ADC) (127/161), and 83.23% (141/161) had advanced stages of CRC at diagnosis (IIIA-IV). Capecitabine treatment regimen distribution was nearly equitable, accounting for 51.55% (83/161) for combination therapy and 48.45% (78/161) for monotherapy. Median age at diagnosis and median tumor size were 65 (56, 73) years and 4.20 [3.00–6.00] cm, respectively. All patients were Caucasian, specifically identified as the Iberian population in Spain (IBS). Only 4.35% of patients (7/161) were found to carry clinically relevant DPYD variants, specifically as heterozygote carriers of the decreased activity DPYD variants rs67376798 and rs56038477. During capecitabine-based treatment, 44.1% (71/161) of patients experienced severe general toxicity. Dose-limiting toxicities included abdominal pain (3.73%; 6/161), diarrhea (8.07%; 13/161), nausea (3.73%; 6/161), and HFS (4.35%; 7/161). No cases of severe stomatitis were reported and 27.33% (44/161) of patients discontinued treatment due to capecitabine toxicity.

### 3.2. Association of Sociodemographic and Clinical Variables with Toxicity and Treatment Suspension

None of the sociodemographic and clinical variables were significantly associated with the overall toxicity related to the capecitabine-based treatment. However, a trend of association with severe overall toxicity was observed in patients with cancer of rectal origin (*p* = 0.057; OR = 1.85; 95% CI = 0.98–3.51; Table S2). With respect to dose-limiting toxicities, severe diarrhea was significantly associated with an alcohol habit (*p* = 0.036; Table S3) and mucinous ADC (*p* = 0.032; OR = 3.67; 95% CI = 1.11–11.91; Table S3). Also, a trend of association between diarrhea and capecitabine in combination was found (*p* = 0.056; OR = 3.42; 95% CI = 1.00–15.72; Table S3). No significant association was found between the occurrence of severe abdominal pain, nausea, or HFS and patients’ sociodemographic and clinical characteristics (*p* > 0.05; Tables S4–S6). 

Capecitabine therapy suspension was significantly associated with PS-1 (*p* = 0.021; OR = 2.42; 95% CI = 1.07–5.44, for 0 vs. 1; Table S7), adjuvant capecitabine monotherapy (*p* = 0.002; OR = 3.08; 95% CI = 1. 50–6.56; Table S7), and advanced age at CRC diagnosis (*p* = 0.038; OR = 1.03; 95% CI = 1.00–1.07; Table S7).

### 3.3. Genotype Distribution

**Figure 2:**
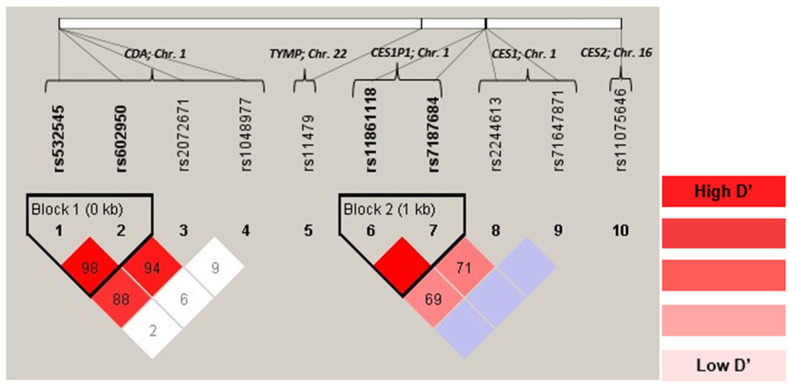
Linkage disequilibrium of selected SNPs.

No significant deviations from HWE were observed in any of the analyzed SNPs (Table S8). Minor allele frequency exceeded 1% for all the SNPs studied (Table S9). LD of the selected SNPs and r2 values are shown in Table S10, and Figure 2 shows the LD plot. The SNPs in CDA gene rs532545, rs602950, and rs2072671 were strongly correlated. According to the obtained r2 values, the CDA rs602950 SNP is a tag-SNP for the SNPs CDA rs532545 (r2 = 0.892 D’ = 0.986) and rs2072671 (r2 = 0.855; D’ = 0.943) (Table S11). The SNPs CES1P1 rs7187684 and rs11861118 were in strong LD (r2 = 0.754; D’ = 0.999). The inferred haplotype frequencies for SNPs in CDA and CES1P1 are shown in Tables S12 and S13.

### 3.4. Association of SNPs in Capecitabine’s Bioactivation Pathway with Toxicity and Treatment Suspension

Overall toxicity was associated with CES1 rs71647871. Patients carrying the CES1 rs71647871-A allele had a higher risk of severe overall toxicity (*p* = 0.044; OR = 8.21; 95% CI = 1.35–157.14, for GG vs. A; Table S14). In regard to dose-limiting toxicities, it was observed that carriers of the CES1 rs71647871-A allele were more likely to develop severe HFS (*p* = 0.030; OR = 11.92; 95% CI = 1.46–73.47, for GG vs. A; Table S15). No association was found between SNPs involved in capecitabine’s bioactivation with the risk of diarrhea, abdominal pain, or nausea (*p* > 0.05 Tables S16–S18). However, a trend was found between severe nausea and TYMP rs11479-TT (*p* = 0.056; OR = 34.00; 95% CI = 1.20–976.66, for CC vs. TT; Table S18).

Treatment suspension was associated with CES1P1 rs7187684 and CDA rs1048977. Specifically, patients carrying the CES1P1 rs7187684-T allele (*p* = 0.024; OR = 2.05; 95% CI = 1.01–4.19, for CC vs. T; Table S19) and the CDA rs1048977-CC genotype (*p* = 0.032; OR = 2.17; 95% CI = 1.07–4.19, for T vs. CC; Table S19) were more likely to discontinue capecitabine-based treatment due to toxicity. None of the other SNPs related to capecitabine’s bioactivation were significantly associated with treatment suspension (Table S19). Multivariate logistic regression analysis adjusted by adjuvant treatment type showed that CDA rs1048977 was the only SNP associated with capecitabine treatment suspension (*p* = 0.030; OR = 2.30; 95% CI = 1.09–5.00, for T vs. CC; Table 2). This association remained significant after applying multiple comparison adjustments (*p* = 0.045; Table 2).

In the haplotype analysis, a trend of association was found between haplotype CES1P1 (rs7187684-rs11861118), TG, and treatment suspension (*p* = 0.05; OR = 2.08; 95% CI = 1.01–4.31, for reference haplotype CA vs. haplotype TG) (Table 3). No further associations were found. Tables S20–S30 show the results of the haplotype association analysis.

## 4. Discussion

Validation of genetic biomarkers beyond DPYD is crucial to ensure the safety of capecitabine-based therapy and to advance personalized medicine. Particularly, the genes related to capecitabine’s bioactivation pathway are of significant interest in this regard. In this study, we found that the CES1 rs71647871-A allele was associated with an increased risk of severe HFS in patients undergoing capecitabine-based treatment. Furthermore, we identified the CDA rs1048977-CC genotype and capecitabine monotherapy as potential predictors of treatment suspension. These findings support the hypothesis suggesting the influence that SNPs (related to capecitabine’s bioactivation pathway) have on drug safety.

The CES gene family is located on chromosome 16 (16q12.2-22.1), which encodes enzymes of the α/β-hydrolase family (CES1 and CES2). CES1 is the most expressed enzyme in humans and plays a crucial role in activating/deactivating numerous substrates [35,36]. CES1 acts as the first step in the bioactivation pathway of capecitabine to 5-FU [17]. The missense variant CES1 rs71647871 (c.428G > A) results in the substitution of the amino acid Glycine with Glutamic acid (p.Gly143Glu) [37]. In our study, an association was found between the CES1 rs71647871-A allele and an increased risk of severe HFS, contrary to the findings by Hamzic et al. (Caucasian; *n* = 111)—the only previous study that analyzed this variant. In this study, no significant association was found between the SNP CES1 rs71647871 and the early onset of capecitabine-related toxicity in patients with solid tumors (*p*-value > 0.05) [25]. Previous studies on different drugs, such as clopidogrel, enalapril, trandolapril, and methylphenidate, have found an association between the presence of the A allele with decreased enzyme activity and reduced drug metabolism [38,39,40,41,42,43,44]. However, for quinapril, which is also bioactivated by CES1, the CES1 rs71647871 SNP was found unrelated to metabolism and drug clearance in healthy individuals [39]. All these findings could suggest that the impact of SNPs in CES1 on enzymatic activity depends on the specific substrate they interact with. Currently, there are no studies evaluating the impact of the CES1 rs71647871 SNP on CES1 enzymatic activity with capecitabine as the substrate. Cell models and prospective validation studies in larger cohorts could clarify the impact of the SNP on capecitabine’s pharmacokinetics and toxicity, respectively.

The highly homologous pseudogene CES1P1 is located in proximity to the CES1 gene [45]. Studies suggest that CES1P1 could have a regulatory impact by affecting the gene expression levels of CES1 and, consequently, influencing the metabolism of its substrates. In particular, the SNP CES1P1 rs7187684 (g.55761039TT > C; Intron) has been associated with quantitative changes in the expression of the CES1 gene, while the SNP CES1P1 rs11861118 (g.55759367A > G; 2KB upstream) has a RegulomeDB score of 3a, indicating that it is likely to play a role in gene regulation [25]. In our study, we found a trend of association between the CES1P1 rs7187684-T allele and an increased risk of treatment discontinuation due to toxicity. Likewise, the CES1P1 haplotype (rs7187684-rs11861118) TG also exhibited a trend of association with treatment discontinuation. Hamzic et al. (Caucasian population; *n* = 144) reported that in patients with solid neoplasms treated with capecitabine-based regimens, the CES1P1 rs7187684-T allele (*p* = 0.012; OR = 6.51; 95% CI = 1.51–28.00; T vs. C) and the CES1P1 rs11861118 g allele (*p* = 0.012; OR = 6.48; 95% CI = 1.50–28.00; A vs. G) were significantly associated with an early onset of toxicity [25].

CDA is an enzyme synthesized in the liver responsible for the deamination of various drugs, including nucleoside analogs widely used in oncology [46]. In the context of capecitabine, CDA is involved in the second step of its bioactivation pathway [17]. The CDA gene, located on chromosome 1 (1p36.12), is highly polymorphic, and the relationships between the genotypes and phenotypes are not entirely clear. The synonymous variant SNP CDA rs1048977 (c.435C > T; p.Thr145Thr) results in a codon substitution that does not change the encoded amino acid Threonine [47]. In this study, it was found that patients with the CDA rs1048977-CC genotype had a higher likelihood of capecitabine treatment suspension due to toxicity. To date, no other studies have associated this SNP with the risk of treatment discontinuation. However, Pellicer et al., (Caucasian population; Spain; *n* = 301) reported that patients with CRC carrying the CDA rs1048977-T allele (*p* = 0.044; OR = 8.62; 95% CI = 1.05–70.24; CC vs. T) presented a higher risk of capecitabine toxicity, particularly hyperbilirubinemia [24]. This discrepancy with our results could be attributed to the differences in sample size, definition and categorization of toxicity events, and the clinical characteristics of the included patients. Although there are no other studies that have assessed the impact of the CDA rs1048977 SNP on capecitabine’s safety outcomes, the existing evidence concerning its influence on CDA enzymatic activity and treatment outcomes, with the antineoplastic agent gemcitabine as the substrate, also presents conflicting results [48,49,50,51,52]. Additionally, it is important to note that the enzymatic activity of CDA could be influenced by both its genetic coding and the specific drug it metabolizes [46,53]. Conducting additional investigations is imperative to elucidate the impact of the CDA rs1048977 SNP on the enzymatic activity and the treatment’s safety outcomes. 

The TYMP gene, located on chromosome 22 (22q13.33), encodes the TP enzyme. TP plays a significant role in capecitabine’s bioactivation and has also been attributed a function in tumorigenesis, promoting angiogenesis and tumor metastasis due to its structural similarity with the platelet-derived endothelial growth factor [54,55]. The missense variant TYMP rs11479 (c.1412C > T) involves the substitution of the amino acid serine with leucine (p.Ser471Leu) outside the C-terminal domain of the protein. This change occurs in a position where serine is not commonly conserved in mammals [20]. In our study, we observed a trend between the presence of the TYMP rs11479-TT genotype and a higher incidence of severe nausea. The available evidence regarding the impact of the TYMP rs11479 SNP on FP-based therapy toxicity presents conflicting results [20,23,56,57]. Jennings et al. reported a significant association between carriers of the TYMP rs11479-T allele and an increased risk of overall toxicity (OR = 2.70; 95% CI = 1.23–5.92; *p* = 0.013, for CC vs. CT/TT), as well as treatment delays due to toxicity events (OR = 2.02; 95% CI = 1.03–4.00; *p* = 0.042, for CC vs. CT/TT), in a sample of 254 patients with CRC of Caucasian origin treated with FP [20]. In contrast, studies conducted by Caronia et al. in 130 patients from a Spanish population with colorectal and breast cancer treated with capecitabine [57], Meulendijks et al. on 185 gastric cancer patients of Dutch origin treated with capecitabine-based regimens [23], and Chen et al. on 198 gastric cancer patients from a Chinese population undergoing capecitabine-based treatment [56] failed to identify significant associations in this context (*p* > 0.050). It is noteworthy that Chen et al. reported a trend of association between the carriers of the TYMP rs11479-T allele and a higher incidence of HFS (*p* = 0.092) and grade 2 anemia (*p* = 0.056), indicating that the T allele carriers of this SNP may be more sensitive to capecitabine compared to C allele carriers [56]. Despite the uncertainty regarding the functional impact and clinical relevance of this SNP on TP activity, previous research has reported that carriers of the TYMP rs11479-T allele exhibit higher levels of TYMP gene expression and mRNA compared to carriers of the G allele [56,58]. The conflicting findings in the available evidence emphasize the need for further research to thoroughly understand the relationship between the TYMP rs11479 SNP and its impact on toxicity in capecitabine-based therapy.

In our investigation, it was observed that patients under capecitabine monotherapy regimens exhibited a higher rate of treatment discontinuation in comparison to those treated with combination regimens. Factors that could account for this observation have been identified. Capecitabine toxicity profiles differ in monotherapy and combination regimens; in particular, combination therapy might be expected to be associated with higher levels of adverse events [59]. However, serious adverse effects such as nausea, HFS, mucositis, and abdominal pain, which often prompt treatment discontinuation, are more prevalent in monotherapy schedules [6,59]. Additionally, patients who are prescribed with capecitabine in monotherapy are predominantly the elderly, and this particular patient cohort presents a higher prevalence of pre-existing health conditions, differences in drug metabolism and elimination, and comorbidities [60]. These factors make them more susceptible to reduced treatment tolerability, increased incidence of toxicity, and potential delays or discontinuation of antineoplastic treatment [61,62]. Of note, the decision to discontinue or modify chemotherapy is complex and is made by the oncologist together with the patient, considering several individual factors such as cancer type and stage, response to treatment, and tolerability [63]. 

The main limitation of our study is the relatively small sample size, which may have limited the statistical power to identify more subtle associations of other SNPs in genes involved in the bioactivation of capecitabine with the safety of antineoplastic therapy. Studies involving larger prospective cohorts could enhance the validity and generalizability of our findings. Furthermore, the study evaluated SNP associations on a specific Caucasian IBS population undergoing adjuvant treatment for CRC; therefore, the results obtained cannot be extrapolated to other ethnicities, treatment regimens, or cancer types. Future research should include diverse patient cohorts to obtain more comprehensive results. Despite these limitations, our study contributes to the growing body of evidence on the role of genetic variability in capecitabine safety. 

Regarding the clinical implications of our findings, the associations found provide valuable insights into the impact of non-DPYD variants, specifically CES1 rs71647871 and CDA s1048977, on the toxicity of capecitabine-based therapy in terms of the hand–foot syndrome (HFS) and treatment suspension due to toxicity. Identifying genetic factors associated with treatment safety contributes to the practice of personalized medicine. Consequently, clinicians should consider their patients’ genetic profiles related to capecitabine’s bioactivation in addition to the currently applied DPYD metabolizer status that is used in clinical practice. This approach has the potential to substantially reduce severe adverse events of toxicity and enhance clinical outcomes in patients.

## 5. Conclusions

Patients with CRC carrying the CDA rs1048977-CC genotype were found to be at a higher risk of discontinuing adjuvant capecitabine-based treatment due to toxicity. Additionally, capecitabine monotherapy exhibited a greater likelihood of treatment suspension. The CES1 rs71647871-A allele was associated with an increased susceptibility to severe HFS. The impact of the SNPs (CES1 rs71647871 and CDA rs1048977) on enzymatic activity appears to depend on which specific drug is used as the substrate, as suggested by the existing body of evidence. However, there are currently no studies available on capecitabine. Therefore, further research is necessary to confirm the effects of these SNPs on enzymatic activity and safety outcomes when capecitabine is the substrate. This would help to clarify our findings and determine whether these SNPs can be considered as potential predictive biomarkers of safety in capecitabine-based therapy.

## Figures and Tables

**Table 1:** Baseline characteristics of 161 patients with CRC included in this study.

Characteristic		*n*	(%)
Sex	Female	62	38.51
	Male	99	61.49
Smoking habit	Current Smoker	32	19.88
	Non-smoker	90	55.90
	Former smoker	39	24.22
Alcohol habit	Current drinker	35	21.74
	Non-drinker	117	72.67
	Former drinker	9	5.59
Age at diagnosis (years)		65 (56–73)	
Tumor location	Colon	95	59.01
	Rectum	66	40.99
Tumor size (cm)		4.20 [3.00–6.00]	
Stage at diagnosis	0-IIC	20	16.77
	IIIA-IV	141	83.23
Histology	ADC	127	78.88
	Mucinous ADC	34	21.12
Grade	High	20	12.42
	Low	141	87.58
PS ECOG	0	116	72.05
	1	35	21.74
	2	10	6.21
Type of capecitabine-based adjuvant treatment	Monotherapy	78	48.45
	Combination	83	51.55
Clinically validated DPYD variants carrier *	Yes	7	4.35
	No	154	95.65
General toxicity	Grade < 3	90	55.9
	Grade ≥ 3	71	44.1
Abdominal pain	Grade < 3	155	96.27
	Grade ≥ 3	6	3.73
Diarrhea	Grade < 3	148	91.93
	Grade ≥ 3	13	8.07
Stomatitis	Grade < 3	161	100
	Grade ≥ 3	0	0.00
Nausea	Grade < 3	155	96.7
	Grade ≥ 3	6	3.73
HFS	Grade < 3	154	95.65
	Grade ≥ 3	7	4.35
Capecitabine-based treatment suspension	Yes	44	27.33
	No	117	72.67

Qualitative variables: frequency (percentage). Quantitative variables: p50 [p25–p75] (nonparametric distribution); ADC: adenocarcinoma; CRC: colorectal cancer; *n*: number; HFS: hand and foot syndrome; * heterozygote carriers of reduced activity DPYD variants: rs67376798 and rs56038477.

**Table 2:** Multivariate regression analysis.

	**Overall Toxicity**		
	**OR (95% CI)**	***p*** **-Value**	p-BH *
CES1 rs71647871 (A)	7.71 (1.25–148.48)	0.062	0.076
Tumor localization (rectum)	1.79 (0.94–3.44)	0.076	0.076
Model *p*-value = 0.010			
	**Treatment Suspension**		
	**OR (95% CI)**	***p*** **-Value**	p-BH *
CES1P1 rs7187684 (T)	2.10 (0.99–4.47)	0.051	0.051
CDA rs1048977 (CC)	2.30 (1.09–5.00)	0.030	0.045
Type of adjuvant treatment (monotherapy)	3.13 (1.49–6.81)	0.003	0.009
Model *p*-value < 0.001			

OR: odds ratio; 95% CI: 95% confidence interval; * *p*-value adjusted with Benjamini–Hochberg.

**Table 3:** Haplotypes of SNPs in gene CES1P1 located in chromosome 16 and associated with treatment suspension (*n* = 161).

H	CES1P1 rs7187684	CES1P1 rs11861118	Frequencies	OR (CI95%)	*p*-Value **
1	C	A	0.8106	1.00	---
2	T	G	0.1584	2.08 (1.01–4.31)	0.050
3	T	A	0.0311	2.98 (0.87–10.22)	0.084
Global haplotype association *p*-value: 0.047					

Gray: most common haplotype (reference); CI 95%: 95% confidence interval. H: haplotype; OR: odds ratio; ** adjusted by stage, type of capecitabine-based adjuvant treatment, and PS ECOG.

## Data Availability

Data are unavailable due to privacy and ethical restrictions.

## Supplementary Materials

The following supporting information can be downloaded at: https://www.mdpi.com/article/10.3390/pharmaceutics15112548/s1, Table S1. Selected SNPs in capecitabine’s bioactivation pathway genes; Table S2. Association of sociodemographic and clinical characteristics with overall toxicity; Table S3. Association of sociodemographic and clinical characteristics with diarrhea; Table S4. Association of sociodemographic and clinical characteristics with abdominal pain; Table S5. Association of sociodemographic and clinical characteristics with nausea; Table S6. Association of sociodemographic and clinical characteristics with HFS; Table S7. Association of sociodemographic and clinical characteristics with treatment suspension; Table S8. Hardy–Weinberg equilibrium analysis of the selected SNPs; Table S9. Minor allele frequency of the selected SNPs; Table S10. Linkage disequilibrium of the selected SNPs; Table S11. Tag-SNP analysis for CDA rs602950, rs532545, and rs2072671; Table S12. Estimation of frequencies for SNP haplotypes in CDA (located on chromosome 1); Table S13. Estimation of frequencies for SNP haplotypes in CES1P1 (located on chromosome 16); Table S14. Association of SNPs in capecitabine’s bioactivation pathway with overall toxicity; Table S15. Association of SNPs in capecitabine’s bioactivation pathway with HFS; Table S16. Association of SNPs in capecitabine’s bioactivation pathway with diarrhea; Table S17. Association of SNPs in capecitabine’s bioactivation pathway with abdominal pain; Table S18. Association of SNPs in capecitabine’s bioactivation pathway with nausea; Table S19. Association of SNPs in capecitabine’s bioactivation pathway with treatment suspension; Table S20. Haplotypes of SNPs in gene CDA located in chromosome 1 and association with overall toxicity; Table S21. Haplotypes of SNPs in gene CDA located in chromosome 1 and association with diarrhea; Table S22. Haplotypes of SNPs in gene CDA located in chromosome 1 and association with abdominal pain; Table S23. Haplotypes of SNPs in gene CDA located in chromosome 1 and association with nausea; Table S24. Haplotypes of SNPs in gene CDA located in chromosome 1 and association with HFS; Table S25. Haplotypes of SNPs in gene CDA located in chromosome 1 and association with treatment suspension; Table S26. Haplotypes of SNPs in gene CES1P1 located in chromosome 16 and association with overall toxicity; Table S27. Haplotypes of SNPs in gene CES1P1 located in chromosome 16 and association with diarrhea; Table S28. Haplotypes of SNPs in gene CES1P1 located in chromosome 16 and association with abdominal pain; Table S29. Haplotypes of SNPs in gene CES1P1 located in chromosome 16 and association with nausea; Table S30. Haplotypes of SNPs in gene CES1P1 located in chromosome 16 and association with HFS.

## Abbreviations

5′dFCR: 5′-deoxy-5-fluorocytidine; 5′dFUR: 5′-deoxy-5-fluorouridine; 5-FU: 5-fluorouracil; 95% CIs: 95% confidence intervals; ADC: adenocarcinoma; CES1: carboxylesterase 1 enzyme; CES1: carboxylesterase 1 gene; CES1P1: carboxylesterase 1 pseudogene 1; CES2: carboxylesterase 2 enzyme; CES2: carboxylesterase 2 gene; CDA: cytidine deaminase enzyme; CDA: cytidine deaminase gene; CRC: colorectal cancer; CTCAE: Common Terminology Criteria for Adverse Events; DPD: dihydropyrimidine dehydrogenase enzyme; DPYD: dihydropyrimidine dehydrogenase gene; FDR: false discovery rate; FP: fluoropyrimidines; H: haplotype; HapB3: haplotype B3; HFS: hand–foot syndrome; HWE: Hardy–Weinberg equilibrium; IBS: Iberian population in Spain; LD: linkage disequilibrium; OR: odds ratio; PCR: polymerase chain reaction; PK: pharmacokinetics; PS: performance status; SNP: single-nucleotide polymorphism; TP: thymidine phosphorylase enzyme; TYMP: thymidine phosphorylase gene; UPP: uridine phosphorylase.
