# Supplementary material for: Association of Single-Nucleotide Polymorphisms in Capecitabine Bioactivation Pathway with Adjuvant Therapy Safety in Colorectal Cancer Patients
Source: Pharmaceutics. 2023 Oct 28;15(11):2548. doi: 10.3390/pharmaceutics15112548 (PMC10675271; doi:10.3390/pharmaceutics15112548)
Supplement: Supplementary file 1 [file pharmaceutics-15-02548-s001.zip › pharmaceutics-2641402-supplementary.pdf]

Table S1. Selected SNPs in capecitabine bioactivation pathway genes.

Gene	SNP rs number	Alleles	Variant/ Gene consequence	Assay ID
CES1	rs2244613	G>T	Intron variant	C__11290377_10
CES1	rs71647871	G>A	Missense variant	-
CES1P1	rs7187684	T>C	Intron variant	C__31071358_20
CES1P1	rs11861118	A>G	2KB upstream variant	-
CES2	rs11075646	C>G	5 primer UTR variant	-
CDA	rs532545	C>T	2KB upstream variant	C___7477400_20
CDA	rs602950	A>G	2KB upstream variant	-
CDA	rs2072671	A>C	Missense variant	C__25472931_20
CDA	rs1048977	C>T	Synonymous variant	C___7477307_30
TYMP	rs11479	C>T	Missense variant	C__11946264_20

- customized assay

Table S2. Association of sociodemographic and clinical characteristics with overall toxicity.

**Characteristic**		**n**	**Overall toxicity**		**χ2**	**p-value**	**Ref. cat.**	**OR**	**CI 95%**
			**Grade ≥ 3 n (%)**	**Grade < 3 n (%)**					
**Sex**	Female	62	30 (48.4)	32 (51.6)	0.751	0.385	-	-	-
	Male	99	41 (41.4)	58 (58.6)					
**Smoking habit**	Smoker	32	15 (46.90)	17 (53.1)	0.763	0.682	-	-	-
	Non-smoker	90	37 (41.10)	53 (58.90)					
	Former smoker	39	19 (48.70)	20 (51.30)					
**Alcohol habit**	Drinker	35	16 (45.70)	19 (54.30)	-	0.959*	-	-	-
	Non-drinker	117	51 (43.60)	66 (56.40)					
	Former drinker	9	4 (44.40)	5 (55.60)					
**Tumor location**	Colon	95	36 (37.90)	59 (62.10)	3.619	**0.057**	Colon	1.85	0.98 - 3.51
	Rectum	66	35 (53.00)	31 (47.00)					
**Stage at diagnosis**	0-IIC	20	7 (35.00)	13 (65.6)	0.767	0.381	-	-	-
	IIIA-IV	141	64 (45.40)	77 (54.60)					
**Histology**	ADC	127	53 (41.70)	74 (58.30)	1.366	0.242	-	-	-
	mucinous ADC	34	18 (52.90)	16 (47.10)					
**Grade**	High	20	7 (35.00)	13 (65.00)	0.767	0.381	-	-	-
	Low	141	64 (45.40)	77 (54.60)					
**PS ECOG**	0	116	52 (44.80)	64 (55.20)	-	1.000*	-	-	-
	1	35	15 (42.90)	20 (57.10)					
	2	10	4 (40.00)	6 (60.00)					
**Type of capecitabine-based adjuvant treatment**	Monotherapy	78	33 (42.30)	45 (57.70)	0.197	0.657	-	-	-
	Combination	83	38 (45.80)	45 (54.20)					
**Treatment line**	1st line	148	64 (43.20)	84 (56.80)	-	0.168*	-	-	-
	2nd line	10	4 (40.00)	6 (60.00)					
	3rd line or +	3	3 (100)	0 (0.00)					
***DPYD* variants carrier**	Yes	7	2 (28.60)	5 (71.40)	-	0.466*	-	-	-
	No	154	69 (44.80)	85 (55.20)					

**Characteristic**	**Overall toxicity**		**p-value [b]**	**OR**	**95% CI**
	**Grade ≥ 3 [p50 (p25-p75)]**	**Grade < 3 (p50 [p25-p75])**			
**Age at diagnosis**	65.00 [56.00 - 72.50]	67.00 [60.00 -75.00]	0.275	-	-
**Tumor size**	4.10 [3.00 - 6.00]	4.40 [3.00 – 6.00]	0.575	-	-

ADC, adenocarcinoma; CRC, colorectal cancer; n: number of patients; OR: odds ratio, PS ECOGE: performance status; Ref. cat.: reference category; 95% CI: 95% confidence interval.
Pearson's Chi-square test
b Logistic regression analysis

* Fisher's exact test

Table S3. Association of sociodemographic and clinical characteristics with diarrhea.

Characteristics		n	Diarrhea Grade ≥ 3 n (%)	Diarrhea Grade < 3 n (%)	χ²	p-value [a]	Ref. cat.	OR	95% CI
**Sex**	Female	62	8 (12.90)	54 (87.10)	3.167	**0.075**	Male	2.78	0.88 - 9.61
	Male	99	5 (5.10)	94 (94.90)					
**Smoking habit**	Smoker	32	1 (3.10)	31 (96.90)	-	0.537*	-	-	-
	Non-smoker	90	8 (8.90)	82 (91.10)					
	Former smoker	39	4 (10.30)	35 (89.70)					
**Alcohol habit**	Drinker	35	0 (0.00)	35 (100)	-	**0.035***	-	-	-
	Non-drinker	117	11 (9.40)	106 (90.60)					
	Former drinker	9	2 (22.20)	7 (77.80)					
**Tumor location**	Colon	95	110 (10.50)	85 (89.50)	1.876	0.170	-	-	-
	Rectum	66	3 (4.50)	63 (95.50)					
**Stage at diagnosis**	0-IIC	20	0 (0.00)	20 (100)	-	0.372*	-	-	-
	IIIA-IV	141	13 (9.20)	128 (90.80)					
**Histology**	ADC	127	7 (5.50)	120 (94.50)	-	**0.032***	ADC	3.67	1.10 - 11.91
	mucinous ADC	34	6 (17.60)	28 (82.40)					
**Grade**	High	20	1 (5.00)	19 (95.00)	-	1.000*	-	-	-
	Low	141	12 (8.50)	129 (91.50)					
**PS ECOG**	0	116	12 (10.30)	104 (89.70)	-	0.372*	-	-	-
	1	35	1 (2.90)	34 (97.10)					
	2	10	0 (0.00)	10 (100)					
**Type of capecitabine-based adjuvant treatment**	Monotherapy	78	3 (3.80)	75 (96.20)	3.644	**0.056**	Monotherapy	3.42	1.00 - 15.72
	Combination	83	10 (12.00)	73 (88.00)					
**Treatment line**	1st line	148	11 (7.40)	137 (92.60)	-	0.161*	-	-	-
	2nd line	10	1 (10.00)	9 (90.00)					
	3rd line or +	3	1 (33.00)	2 (66.70)					
***DPYD* variants carrier**	Yes	7	0 (0.00)	7 (100)	-	1.000*	-	-	-
	No	154	13 (8.40)	141 (91.60)					

Characteristic	Diarrhea Grade ≥ 3 [P_{50} (P_{25}-P_{75})]	Diarrhea Grade < 3 (P_{50} [P_{25}-P_{75}])	p-value [b]	OR	95% CI
**Age at diagnosis**	65.00 [55.00 - 72.00]	67.00 [56.75 -74.25]	0.191	-	-
**Tumor size**	3.50 [2.50 - 4.10]	4.40 [3.05 – 6.00]	0.154	-	-

ADC, adenocarcinoma; CRC, colorectal cancer; n: number of patients; OR: odds ratio, PS ECOGE: performance status; Ref. cat.: reference category; 95% CI: 95% confidence interval.
a Pearson's Chi-square test
b Logistic regression analysis
* Fisher's exact test

Table S4. Association of sociodemographic and clinical characteristics with abdominal pain.

Characteristics		n	Abdominal pain Grade ≥ 3 n (%)	Abdominal pain Grade < 3 n (%)	χ²	p-value [a]	Ref. cat.	OR	95% CI
**Sex**	Female	62	3 (4.80)	59 (95.20)	-	0.676*	-	-	-
	Male	99	3 (3.00)	96 (97.00)					
**Smoking habit**	Smoker	32	2 (6.20)	30 (93.80)	-	0.330*	-	-	-
	Non-smoker	90	4 (4.40)	86 (95.60)					
	Former smoker	39	0 (0.00)	39 (100)					
**Alcohol habit**	Drinker	35	0 (0.00)	35 (100)	-	0.533*	-	-	-
	Non-drinker	117	6 (5.10)	111 (94.90)					
	Former drinker	9	0 (0.00)	9 (100)					

**Tumor location**	Colon	95	3 (3.20)	92 (96.80)	-	0.689*	-	-	-
	Rectum	66	3 (4.50)	63 (95.50)					
**Stage at diagnosis**	0-IIC	20	1 (5.00)	19 (95.00)	-	0.554*	-	-	-
	IIIA-IV	141	5 (3.50)	136 (96.50)					
**Histology**	ADC	127	5 (3.90)	122 (96.10)	-	1.000*	-	-	-
	mucinous ADC	34	1 (2.90)	33 (97.10)					
**Grade**	High	20	1 (5.00)	19 (95.00)	-	0.554*	-	-	-
	Low	141	5 (3.50)	136 (96.50)					
**PS ECOG**	0	116	3 (2.60)	113 (97.40)	-	0.154*	-	-	-
	1	35	2 (5.70)	33 (94.30)					
	2	10	1 (10.00)	9 (90.00)					
**Type of capecitabine-based adjuvant treatment**	Monotherapy	78	3 (3.80)	75 (96.20)	-	1.000*	-	-	-
	Combination	83	3 (3.60)	80 (96.40)					
**Treatment line**	1st line	148	5 (3.40)	143 (96.60)	-	0.401*	-	-	-
	2nd line	10	1 (10.00)	9 (90.00)					
	3rd line or +	3	0 (0.00)	3 (100)					
***DPYD* variants carrier**	Yes	7	0 (0.00)	7 (100)	-	1.000*	-	-	-
	No	154	6 (3.90)	148 (96.10)					

**Characteristic**	**Abdominal pain**		**p-value [b]**	**OR**	**95% CI**
	**Grade ≥ 3 [P_{50} (P_{25}-P_{75})]**	**Grade < 3 (P_{50} [P_{25}-P_{75}])**			
**Age at diagnosis**	69.00 [57.00 - 81.00]	66.00 [57.00 -73.50]	0.463	-	-
**Tumor size**	3.50 [2.62 - 7.75]	4.25 [3.00 – 6.00]	0.700	-	-

ADC, adenocarcinoma; CRC, colorectal cancer; n: number of patients; OR: odds ratio, PS ECOGE: performance status; Ref. cat.: reference category; 95% CI: 95% confidence interval.
a Pearson's Chi-square test
b Logistic regression analysis
* Fisher's exact test

Table S5. Association of sociodemographic and clinical characteristics with nausea.

**Characteristics**		**n**	**Nausea**		**χ^2**	**p-value**	**Ref. cat.**	**OR**	**95% CI**
			**Grade ≥ 3 n (%)**	**Grade < 3 n (%)**					
**Sex**	Female	62	4 (6.50)	58 (93.50)	-	0.205*	-	-	-
	Male	99	2 (2.00)	97 (98.00)					
**Smoking habit**	Smoker	32	1 (3.10)	31 (96.90)	-	0.855*	-	-	-
	Non-smoker	90	3 (3.30)	87 (96.70)					
	Former smoker	39	2 (5.10)	37 (94.90)					
**Alcohol habit**	Drinker	35	0 (0.00)	35 (100)	-	0.533*	-	-	-
	Non-drinker	117	6 (5.10)	111 (94.90)					
	Former drinker	9	0 (0.00)	9 (100)					
**Tumor location**	Colon	95	3 (3.20)	92 (96.80)	-	0.689*	-	-	-
	Rectum	66	3 (4.50)	63 (95.50)					
**Stage at diagnosis**	0-IIC	20	1 (5.00)	19 (95.00)	-	0.554*	-	-	-
	IIIA-IV	141	5 (3.50)	136 (96.50)					
**Histology**	ADC	127	4 (3.10)	123 (96.90)	-	0.607*	-	-	-
	mucinous ADC	34	2 (5.90)	32 (94.10)					
**Grade**	High	20	0 (0.00)	20 (100)	-	1.000*	-	-	-
	Low	141	6 (4.30)	135 (95.70)					
**PS ECOG**	0	116	5 (4.30)	111 (95.70)	-	1.000*	-	-	-
	1	35	1 (2.90)	34 (97.10)					
	2	10	0 (0.00)	10 (100)					
**Type of capecitabine-**	Monotherapy	78	2 (2.60)	76 (97.40)	-	0.682*	-	-	-
	Combination	83	4 (4.80)	79 (95.20)					

**based adjuvant treatment**									
**Treatment line**	1st line	148	5 (3.40)	143 (96.60)	-	0.150*	-	-	-
	2nd line	10	0 (0.00)	10 (100)					
	3rd line or +	3	1 (33.30)	2 (66.70)					
***DPYD* variants carrier**	Yes	7	0 (0.00)	7 (100)		-	1.000*	-	-
	No	154	6 (3.90)	148 (96.10)					

**Characteristics**	**Nausea**		**p-value** [b]	**OR**	**95% CI**
	**Grade ≥ 3 [P_{50} (P_{25}-P_{75})]**	**Grade < 3 (P_{50} [P_{25}-P_{75}])**			
**Age at diagnosis**	69.00 [58.50 - 72.75]	66.00 [57.00 -74.00]	0.895	-	-
**Tumor size**	3.25 [2.50 - 4.07]	4.35 [3.00 – 6.00]	0.377	-	-

ADC, adenocarcinoma; CRC, colorectal cancer; n: number of patients; OR: odds ratio, PS ECOGE: performance status; Ref. cat.: reference category; 95% CI: 95% confidence interval.
b Logistic regression analysis
* Fisher's exact test

Table S6. Association of sociodemographic and clinical characteristics with HFS.

**Characteristics**		**n**	**HFS**		**χ²**	**p-value**	**Ref. cat.**	**OR**	**95% CI**
			**Grade ≥ 3 n (%)**	**Grade < 3 n (%)**					
**Sex**	Female	62	2 (3.20)	60 (96.80)	-	0.707*	-	-	-
	Male	99	5 (5.10)	94 (94.90)					
**Smoking habit**	Smoker	32	0 (0.00)	32 (100)	-	0.386*	-	-	-
	Non-smoker	90	4 (4.40)	86 (95.60)					
	Former smoker	39	3 (7.70)	36 (92.30)					
**Alcohol habit**	Drinker	35	0 (0.00)	35 (100)	-	0.462*	-	-	-
	Non-drinker	117	7 (6.00)	110 (94.00)					
	Former drinker	9	0 (0.00)	9 (100)					
**Tumor location**	Colon	95	3 (3.20)	92 (96.80)	-	0.446*	-	-	-
	Rectum	66	4 (6.10)	62 (93.90)					
**Stage at diagnosis**	0-IIC	20	1 (5.00)	19 (95.00)	-	1.000*	-	-	-
	IIIA-IV	141	6 (4.30)	135 (95.70)					
**Histology**	ADC	127	6 (4.70)	121 (95.30)	-	1.000*	-	-	-
	mucinous ADC	34	1 (2.90)	33 (97.10)					
**Grade**	High	20	1 (5.00)	19 (95.00)	-	1.000*	-	-	-
	Low	141	6 (4.30)	135 (95.70)					
**PS ECOG**	0	116	4 (3.40)	112 (96.60)	-	**0.074***	1	1.21	0.17 - 24.18
	1	35	1 (2.90)	34 (97.10)				1.00	-
	2	10	2 (20.00)	8 (80.00)				8.50	0.72 - 196.95
**Type of capecitabine-based adjuvant treatment**	Monotherapy	78	5 (6.40)	73 (93.60)	-	0.265*	-	-	-
	Combination	83	2 (2.40)	81 (97.60)					
**Treatment line**	1st line	148	7 (4.70)	141 (95.30)	-	1.000*	-	-	-
	2nd line	10	0 (0.00)	10 (100)					
	3rd line or +	3	0 (0.00)	3 (100)					
***DPYD* variants carrier**	Yes	7	0 (0.00)	7 (100)	-	1.000*	-	-	-
	No	154	7 (4.50(	147 (95.50)					

**Characteristics**	**HFS**		**p-value** [b]	**OR**	**95% CI**
	**Grade ≥ 3 [P_{50} (P_{25}-P_{75})]**	**Grade < 3 (P_{50} [P_{25}-P_{75}])**			
**Age at diagnosis**	69.00 [61.50 - 73.00]	66.00 [57.00 -74.00]	0.734	-	-
**Tumor size**	3.20 [2.60 - 5.75]	4.30 [3.00 – 6.00]	0.967	-	-

ADC, adenocarcinoma; CRC, colorectal cancer; HFS: hand-foot syndrome; n: number of patients; OR: odds ratio, PS ECOGE: performance status; Ref. cat.: reference category; 95% CI: 95% confidence interval.

b Logistic regression analysis
* Fisher's exact test

Table S7. Association of sociodemographic and clinical characteristics with treatment suspension.

Characteristics		n	Treatment suspension Si n (%)	Treatment suspension No n (%)	χ^2	p-value [a]	Ref. cat.	OR	95% CI
**Sex**	Female	62	20 (32.30)	42 (67.70)	1.233	0.266	-	-	-
	Male	99	24 (24.20)	75 (75.80)					
**Smoking habit**	Smoker	32	8 (25.00)	24 (75.00)	0.765	0.681	-	-	-
	Non-smoker	90	27 (30.00)	63 (70.00)					
	Former smoker	39	9 (23.10)	30 (76.90)					
**Alcohol habit**	Drinker	35	8 (22.90)	27 (77.10)	-	0.773*	-	-	-
	Non-drinker	117	34 (29.10)	83 (70.90)					
	Former drinker	9	2 (22.20)	7 (77.80)					
**Tumor location**	Colon	95	29 (30.50)	66 (69.50)	1.192	0.274	-	-	-
	Rectum	66	15 (22.70)	51 (77.30)					
**Stage at diagnosis**	0-IIC	20	6 (30.00)	14 (70.00)	0.082	0.774	-	-	-
	IIIA-IV	141	38 (27.00)	103 (73.00)					
**Histology**	ADC	127	33 (74.00)	94 (26.00)	0.547	0.459	-	-	-
	mucinous ADC	34	11 (67.60)	23 (32.40)					
**Grade**	High	20	6 (30.00)	14 (70.00)	0.082	0.774	-	-	-
	Low	141	38 (27.00)	103 (73.00)					
**PS ECOG**	0	116	25 (21.60)	91 (78.40)	-	**0.021***	0	1.00	-
	1	35	14 (40.00)	21 (60.00)				**2.42**	**1.07 - 5.44**
	2	10	5 (50.00)	5 (50.00)				3.64	0.94 - 14.07
**Type of capecitabine-based adjuvant treatment**	Monotherapy	78	30 (38.50)	48 (61.50)	9.441	**0.002**	Combination	3.08	1.50 - 6.56
	Combination	83	14 (16.90)	69 (83.10)					
**Treatment line**	1st line	148	40 (27.00)	108 (73.00)	-	0.295*	-	-	-
	2nd line	10	2 (20.00)	8 (80.00)					
	3rd line or +	3	2 (66.70)	1 (33.30)					
***DPYD* variants carrier**	Yes	7	2 (28.60)	5 (71.40)	-	1.000*	-	-	-
	No	154	42 (27.30)	112 (72.70)					

Characteristics	Treatment suspension Si [P_{50} (P_{25}-P_{75})]	Treatment suspension No (P_{50} [P_{25}-P_{75}])	p-value [b]	OR	95% CI
**Age at diagnosis**	70.50 [60.75 - 76.00]	65.00 [57.00 -73.00]	**0.038**	1.03	1.00 - 1.07
**Tumor size**	4.00 [2.70 - 6.00]	4.40 [3.17 – 6.00]	0.775	-	-

ADC, adenocarcinoma; CRC, colorectal cancer; n: number of patients; OR: odds ratio, PS ECOGE: performance status; Ref. cat.: reference category; 95% CI: 95% confidence interval.
a Pearson's Chi-square test
b Logistic regression analysis
* Fisher's exact test

Table S8. Hardy-Weinberg Equilibrium Analysis of the selected SNPs.

Chr	SNP	Minor Allele	Major Allele	Genotype counts	Observed heterozygosity	Expected heterozygosity	p-value
1	rs532545	T	C	24/69/68	0.4286	0.4627	0.394
1	rs602950	G	A	23/66/72	0.4099	0.4537	0.226
1	rs2072671	C	A	23/70/68	0.4348	0.4609	0.495
1	rs1048977	T	C	18/59/84	0.3665	0.4160	0.132
16	rs2244613	G	T	4/48/109	0.2981	0.2873	0.788

16	rs71647871	A	G	0/7/154	0.0435	0.0425	1.000
16	rs7187684	T	C	4/53/104	0.3292	0.3071	0.451
16	rs11861118	G	A	2/47/112	0.2919	0.2666	0.373
16	rs11075646	G	C	3/27/131	0.1677	0.1840	0.216
22	rs11479	T	C	2/19/140	0.1180	0.1327	0.180

Chr: chromosome; SNP: single nucleotide polymorphism

Table S9. Minor allele frequency of the selected SNPs.

Chr	SNP	Minor allele	Major Allele	MAF
1	rs532545	T	C	0.3634
1	rs602950	G	A	0.3478
1	rs2072671	C	A	0.3602
1	rs1048977	T	C	0.2950
16	rs2244613	G	T	0.1739
16	rs71647871	A	G	0.0217
16	rs7187684	T	C	0.1894
16	rs11861118	G	A	0.1584
16	rs11075646	G	C	0.1025
22	rs11479	T	C	0.0714

Table S10. Linkage disequilibrium of the selected SNPs.

Chr	BP	SNP	Chr	BP	SNP	R^2	D
**1**	**20588679**	**rs532545**	**1**	**20589038**	**rs602950**	**0.892538**	**0.986**
1	20588679	rs532545	1	20589208	rs2072671	0.774629	0.889
**1**	**20589038**	**rs602950**	**1**	**20589208**	**rs2072671**	**0.855574**	**0.943**
16	55810697	rs2244613	16	55761039	rs7187684	0.463807	0.719
16	55810697	rs2244613	16	55759367	rs11861118	0.398762	0.697
16	55761039	rs7187684	16	55759367	rs11861118	0.754666	0.999

BP: Base-pair physical position ; Chr: chromosome; SNP: single nucleotide polymorphism

Table S11. Tag-SNP analysis for CDA rs602950, rs532545 and rs2072671.

SNP	Chr	BP	NTag	Left	Right	KBSpan	Tags
rs532545	1	0	1	0	0	0	rs602950
rs602950	1	0	2	0	0	0	rs532545|rs2072671
rs2072671	1	0	1	0	0	0	rs602950

BP: Base-pair physical position ; Chr: chromosome; SNP: single nucleotide polymorphism

Table S12. Estimation of frequencies for SNP haplotypes in CDA (located on chromosome 1).

**H**	***CDA* rs532545**	***CDA* rs602950**	***CDA* rs2072671**	**Total**	**Cumulative frequency**	**Abdominal pain**		**Diarrhea**		**Nausea**		**HFS**		**Overall toxicity**		**Treatment suspension**	
						**Grade ≥ 3**	**Grade < 3**	**Grade ≥ 3**	**Grade < 3**	**Grade ≥ 3**	**Grade < 3**	**Grade ≥ 3**	**Grade < 3**	**Grade ≥ 3**	**Grade < 3**	**Yes**	**No**
1	C	T	A	0.6085	0.6085	0.5833	0.6094	0.5	0.618	0.5833	0.6094	0.8571	0.5972	0.6123	0.6055	0.6023	0.6109
2	T	C	C	0.3352	0.9436	0.3333	0.3352	0.4615	0.3241	0.3333	0.3352	0.1429	0.3439	0.3306	0.3388	0.3523	0.3288

3	C	T	C	0.0251	0.9687	NA	0.026	0.0385	0.0239	NA	0.0261	NA	0.0262	0.0426	0.0112	0.0341	0.0216
4	T	T	A	0.0186	0.9874	0.0833	0.0161	NA	0.0203	0.0833	0.0161	NA	0.0195	0.007	0.0278	0.0114	0.0214
*	T	C	A	0.0095	0.9969	NA	0.0099	NA	0.0104	NA	0.0099	NA	0.01	0.0074	0.0112	NA	0.0131
*	C	C	A	0.0031	1	NA	0.0032	NA	0.0034	NA	0.0032	NA	0.0032	NA	0.0056	NA	0.0043

Gray: most frequent haplotype
* : rare haplotype, defined as an haplotype with a frequency < 0.01.
H: haplotype; HFS: hand-foot syndrome; NA: not available

Table S13. Estimation of frequencies for SNP haplotypes in *CES1P1* (located on chromosome 16).

H	***CES1P1* rs7187684**	***CES1P1* rs11861118**	**Total**	**Cumulative frequency**	**Abdominal pain**		**Diarrhea**		**Nausea**		**HFS**		**Overall toxicity**		**Treatment suspension**	
					**Grade ≥ 3**	**Grade < 3**	**Grade ≥ 3**	**Grade < 3**	**Grade ≥ 3**	**Grade < 3**	**Grade ≥ 3**	**Grade < 3**	**Grade ≥ 3**	**Grade < 3**	**Yes**	**No**
1	C	A	0.8106	0.8106	0.6667	0.8161	0.9231	0.8007	1	0.8032	0.8571	0.8084	0.8099	0.8111	0.8099	0.8111
2	T	G	0.1584	0.9689	0.2500	0.1548	0.0769	0.1655	NA	0.1645	0.0714	0.1623	0.1549	0.1611	0.1549	0.1611
3	T	A	0.0311	1	0.0833	0.0290	NA	0.0338	NA	0.0323	0.0714	0.0292	0.0352	0.0278	0.0352	0.0278

Gray: most frequent haplotype
H: haplotype; HFS: hand-foot syndrome; NA: not available

Table S14. Association of SNPs in capecitabine bioactivation pathway with overall toxicity.

**Gene**	**SNP**	**Genotype**	**n**	**Overall Toxicity: Grade ≥ 3 n (%)**	**Overall Toxicity: Grade < 3 n (%)**	**χ^2**	**p-value [a]**	**Ref. cat.**	**OR**	**95% CI**
CES1	rs2244613	GG	4	1 (25.00)	3 (75.00)					
		GT	48	21 (43.80)	27 (56.20)	-	0.852*	-	-	-
		TT	109	49 (45.00)	60 (55.00)					
		G	52	22 (42.30)	30 (57.70)	0.100	0.751	-	-	-
		T	157	70 (44.60)	87 (55.40)	-	0.630*	-	-	-
	rs71647871	GG	154	65 (42.20)	89 (57.80)				1.00	-
		AG	7	6 (85.70)	1 (14.30)	-	**0.044***	GG	8.21	1.35 - 157.14
		AA	0	0 (0.00)	0 (0.00)				-	-
		G	-	-	-	-	-	-	-	-
		A	7	6 (85.70)	1 (14.30)	-	**0.044***	GG	8.21	1.35 - 157.14
CES1P1	rs7187684	CC	104	46 (44.20)	58 (55.80)					
		CT	53	23 (43.40)	30 (56.60)	-	1.000*	-	-	-
		TT	4	2 (50.00)	2 (50.00)					
		C	157	69 (43.90)	88 (56.10)	-	1.000*	-	-	-
		T	57	25 (43.90)	32 (56.10)	0.002	0.963	-	-	-
	rs11861118	AA	112	50 (44.60)	62 (55.40)					
		AG	47	20 (42.60)	27 (57.40)	-	0.931*	-	-	-
		GG	2	1 (50.00)	1 (50.00)					
		A	159	70 (44.00)	89 (56.00)	-	1.000*	-	-	-
		G	49	21 (42.90)	28 (57.10)	0.044	0.833	-	-	-
CES2	rs11075646	CC	131	57 (43.50)	74 (56.50)					
		CG	27	13 (48.10)	14 (51.90)	-	0.865*	-	-	-
		GG	3	1 (33.30)	2 (66.70)					
		C	158	70 (44.30)	88 (55.70)	-	1.000*	-	-	-
		G	30	14 (46.70)	16 (53.30)	0.098	0.753	-	-	-
CDA	rs602950	AA	72	30 (41.70)	42 (58.30)					
		AG	66	34 (51.50)	32 (48.50)	3.387	0.183	-	-	-
		GG	23	7 (30.40)	16 (69.60)					
		A	138	64 (46.40)	74 (53.60)	2.032	0.154	-	-	-

		G	89	41 (46.10)	48 (53.90)	0.312	0.576	-	-	-
	rs1048977	CC	84	42 (50.00)	42 (50.00)	5.531	0.062	CC	1.00	-
		CT	59	19 (32.20)	40 (67.80)				0.47	0.23 - 0.94
		TT	18	10 (55.60)	8 (44.40)				1.25	0.44 - 3.57
		C	143	61 (42.70)	82 (57.30)	1.078	0.298	-	-	-
		T	77	29 (37.70)	48 (62.30)	2.480	0.115	-	-	-
TYMP	rs11479	TT	2	2 (100)	0 (0.00)	-	0.291*	-	-	-
		CT	19	7 (36.80)	12 (63.20)					
		CC	140	62 (44.30)	78 (55.70)					
		T	21	9 (42.90)	12 (57.10)	0.015	0.902	-	-	-
		C	159	69 (43.40)	90 (56.60)	-	0.192*	-	-	-

n: number of patients; OR: odds ratio; Ref. cat.: reference category; SNP: Single nucleotide polymorphism; 95% CI: 95% confidence interval.
a: Pearson's Chi-square test
* Fisher's exact test

Table S15. Association of SNPs in capecitabine bioactivation pathway with HFS.

Gene	SNP	Genotype	n	HFS Grade ≥ 3 n (%)	HFS Grade < 3 n (%)	χ^2	p-value [a]	Ref. cat.	OR	95% CI
CES1	rs2244613	GG	4	0 (0.00)	4 (100)	-	0.729*	-	-	-
		GT	48	1 (2.10)	47 (97.90)					
		TT	109	6 (5.50)	103 (94.50)					
		G	52	1 (1.90)	51 (98.10)	-	0.430*	-	-	-
		T	157	7 (4.50)	150 (95.50)	-	1.000*	-	-	-
	rs71647871	GG	154	5 (3.20)	149 (96.80)	-	**0.030***	GG	11.92	1.46 - 73.47
		AG	7	2 (28.60)	5 (71.40)					
		AA	0	0 (0.00)	0 (0.00)					
		G	-	-	-	-	-	-	-	-
		A	7	2 (28.60)	5 (71.40)	-	**0.030***	GG	11.92	1.46 - 73.47
CES1P1	rs7187684	CC	104	5 (4.80)	99 (95.20)	-	1.000*	-	-	-
		CT	53	2 (3.80)	51 (96.20)					
		TT	4	0 (0.00)	4 (100)					
		C	157	7 (4.50)	150 (95.50)	-	1.000*	-	-	-
		T	57	2 (3.50)	55 (96.50)	-	1.000*	-	-	-
	rs11861118	AA	112	6 (5.40)	106 (94.60)	-	0.702*	-	-	-
		AG	47	1 (2.10)	46 (97.90)					
		GG	2	0 (0.00)	2 (100)					
		A	159	7 (4.40)	152 (95.60)	-	1.000*	-	-	-
		G	49	1 (2.00)	48 (98.00)	-	0.676*	-	-	-
CES2	rs11075646	CC	131	6 (4.60)	125 (95.40)	-	1.000*	-	-	-
		CG	27	1 (3.70)	26 (96.30)					
		GG	3	0 (0.00)	3 (100)					
		C	158	7 (4.4)	151 (95.60)	-	1.000*	-	-	-
		G	30	1 (3.30)	29 (96.70)	-	1.000*	-	-	-
CDA	rs602950	AA	72	5 (6.90)	67 (93.10)	-	0.429*	-	-	-
		AG	66	2 (3.00)	64 (97.00)					
		GG	23	0 (0.00)	23 (100)					
		A	138	7 (5.10)	131 (94.90)	-	0.594*	-	-	-
		G	89	2 (2.20)	87 (97.80)	-	0.243*	-	-	-
	rs1048977	CC	84	6 (7.10)	78 (92.90)	-	0.088*	-	-	-
		CT	59	0 (0.00)	59 (100)					
		TT	18	1 (5.60)	17 (94.40)					
		C	143	6 (4.20)	137 (95.80)	-	0.571*	-	-	-
		T	77	1 (1.30)	76 (98.70)	-	0.119*	-	-	-
TYMP	rs11479	TT	2	0 (0.00)	2 (100)	-	0.631*	-	-	-
		CT	19	1 (5.30)	18 (94.70)					
		CC	140	6 (4.30)	134 (95.70)					
		T	21	1 (4.80)	20 (95.20)	-	1.000*	-	-	-
		C	159	7 (4.40)	152 (95.60)	-	1.000*	-	-	-

HFS: hand-foot syndrome; n: number of patients; OR: odds ratio; Ref. cat.: reference category; SNP: Single nucleotide polymorphism; 95% CI: 95% confidence interval.
a Pearson's Chi-square test
* Fisher's exact test

Table S16. Association of SNPs in capecitabine bioactivation pathway with diarrhea.

Gene	SNP	Genotype	n	Diarrhea		χ^2	p-value [a]	Ref. cat.	OR	95% CI
				Grade ≥ 3 n (%)	Grade < 3 n (%)					
CES1	rs2244613	GG	4	0 (0.00)	4 (100)	-	1.000*	-	-	-
		GT	48	4 (8.30)	44 (91.70)					
		TT	109	9 (8.30)	100 (91.70)					
		G	52	4 (7.70)	48 (92.30)	-	1.000*	-	-	-
		T	157	13 (8.30)	144 (91.70)	-	1.000*	-	-	-
	rs71647871	GG	154	13 (8.40)	141 (91.60)	-	1.000*	-	-	-
		AG	7	0 (0.00)	7 (100)					
		AA	0	0 (0.00)	0 (0.00)					
		G	-	-	-	-	-	-	-	-
		A	7	0 (0.00)	7 (100)	-	1.000*	-	-	-
CES1P1	rs7187684	CC	104	11 (10.60)	93 (89.40)	-	0.445*	-	-	-
		CT	53	2 (3.80)	51 (96.20)					
		TT	4	0 (0.00)	4 (100)					
		C	157	13 (8.30)	144 (91.70)	-	1.000*	-	-	-
		T	57	2 (3.50)	55 (96.50)	-	0.140*	-	-	-
	rs11861118	AA	112	11 (9.80)	101 (90.20)	-	0.449*	-	-	-
		AG	47	2 (4.30)	45 (95.70)					
		GG	2	0 (0.00)	2 (100)					
		A	159	13 (8.20)	146 (91.80)	-	1.000*	-	-	-
		G	49	2 (4.10)	47 (95.90)	-	0.347*	-	-	-
CES2	rs11075646	CC	131	9 (6.90)	122 (93.10)	-	0.409*	-	-	-
		CG	27	4 (14.80)	23 (85.20)					
		GG	3	0 (0.00)	3 (100)					
		C	158	13 (8.20)	145 (91.80)	-	1.000*	-	-	-
		G	30	4 (13.30)	26 (86.70)	-	0.265*	-	-	-
CDA	rs602950	AA	72	4 (5.60)	68 (94.40)	-	0.445*	-	-	-
		AG	66	6 (9.10)	60 (90.90)					
		GG	23	3 (13.00)	20 (87.00)					
		A	138	10 (7.20)	128 (92.80)	-	0.401*	-	-	-
		G	89	9 (10.10)	80 (89.90)	1.113	0.291	-	-	-
	rs1048977	CC	84	9 (10.70)	75 (89.30)	-	0.547*	-	-	-
		CT	59	3 (5.10)	56 (94.90)					
		TT	18	1 (5.60)	17 (94.40)					
		C	143	12 (8.40)	131 (91.60)	-	1.000*	-	-	-
		T	77	4 (5.20)	73 (94.80)	1.648	0.199	-	-	-
TYMP	rs11479	TT	2	0 (0.00)	2 (100)	-	1.000*	-	-	-
		CT	19	1 (5.30)	18 (94.70)					
		CC	140	12 (8.60)	128 (91.40)					
		T	21	1 (4.80)	20 (95.20)	-	1.000*	-	-	-
		C	159	13 (8.20)	146 (91.80)	-	1.000*	-	-	-

n: number of patients; OR: odds ratio; Ref. cat.: reference category; SNP: Single nucleotide polymorphism; 95% CI: 95% confidence interval.

a Pearson's Chi-square test

* Fisher's exact test

Table S17. Association of SNPs in capecitabine bioactivation pathway with abdominal pain.

Gene	SNP	Genotype	n	Abdominal pain		χ^2	p-value [a]	Ref. cat.	OR	95% CI
				Grade ≥ 3 n (%)	Grade < 3 n (%)					
CES1	rs2244613	GG	4	1 (25.00)	3 (75.00)	-	0.151*	-	-	-
		GT	48	1 (2.10)	47 (97.90)					
		TT	109	4 (3.70)	105 (96.30)					
		G	52	2 (3.80)	50 (96.20)	-	1.000*	-	-	-
		T	157	5 (3.20)	152 (96.80)	-	0.142*	-	-	-
	rs71647871	GG	154	6 (3.90)	148 (96.10)	-	1.000*	-	-	-
		AG	7	0 (0.00)	7 (100)					
		AA	0	0 (0.00)	0 (0.00)					
		G	-	-	-	-	-	-	-	-
		A	7	0 (0.00)	7 (100)	-	1.000*	-	-	-
CES1P1	rs7187684	CC	104	3 (2.90)	101 (97.10)	-	0.156*	-	-	-

		CT	53	2 (3.80)	51 (96.20)					
		TT	4	1 (25.00)	3 (75.00)					
		C	157	5 (3.20)	152 (96.80)	-	0.142*	-	-	-
		T	57	3 (5.30)	54 (94.70)	-	0.667*	-	-	-
	rs11861118	AA	112	4 (3.60)	108 (96.40)	-	0.081*	AA	1.00	-
		AG	47	1 (2.10)	46 (97.90)				0.58	0.02 - 4.10
		GG	2	1 (50.00)	1 (50.00)				27.00	0.95 - 776.25
		A	159	5 (3.10)	154 (96.90)	-	0.073*	A	30.80	1.11 - 861.34
		G	49	2 (4.10)	47 (95.90)	-	1.000*	-	-	-
CES2	rs11075646	CC	131	5 (3.80)	126 (96.20)	-	1.000*	-	-	-
		CG	27	1 (3.70)	26 (96.30)					
		GG	3	0 (0.00)	3 (100)					
		C	158	6 (3.80)	152 (96.20)	-	1.000*	-	-	-
		G	30	1 (3.30)	29 (96.70)	-	1.000*	-	-	-
CDA	rs602950	AA	72	2 (2.80)	70 (97.20)	-	0.519*	-	-	-
		AG	66	4 (6.10)	62 (93.90)					
		GG	23	0 (0.00)	23 (100)					
		A	138	6 (4.30)	132 (95.70)	-	0.595*	-	-	-
		G	89	4 (4.50)	85 (95.50)	-	0.692*	-	-	-
	rs1048977	CC	84	3 (3.60)	81 (96.40)	-	0.194*	-	-	-
		CT	59	1 (1.70)	58 (98.30)					
		TT	18	2 (11.10)	16 (88.90)					
		C	143	4 (2.80)	139 (97.20)	-	0.135*	-	-	-
		T	77	3 (3.90)	74 (96.10)	-	1.000*	-	-	-
TYMP	rs11479	TT	2	0 (0.00)	2 (100)	-	0.573*	-	-	-
		CT	19	1 (5.30)	18 (94.70)					
		CC	140	5 (3.60)	135 (96.40)					
		T	21	1 (4.80)	20 (95.20)	-	0.573*	-	-	-
		C	159	6 (3.80)	153 (96.20)	-	1.000*	-	-	-

n: number of patients; OR: odds ratio; Ref. cat.: reference category; SNP: Single nucleotide polymorphism; 95% CI: 95% confidence interval.

a Pearson's Chi-square test

* Fisher's exact test

Table S18. Association of SNPs in capecitabine bioactivation pathway with nausea.

Gene	SNP	Genotype	n	Nausea Grade ≥ 3 n (%)	Nausea Grade < 3 n (%)	χ^2	p-value [a]	Ref. cat.	OR	95% CI
CES1	rs2244613	GG	4	0 (0.00)	4 (100)	-	0.295*	-	-	-
		GT	48	0 (0.00)	48 (100)					
		TT	109	6 (5.50)	103 (94.50)					
		G	52	0 (0.00)	52 (100)	-	0.178*	-	-	-
		T	157	6 (3.80)	151 (96.20)	-	1.000*	-	-	-
	rs71647871	GG	154	6 (3.90)	148 (96.10)	-	1.000*	-	-	-
		AG	7	0 (0.00)	7 (100)					
		AA	0	0 (0.00)	0 (0.00)					
		G	-	-	-	-	-	-	-	-
		A	7	0 (0.00)	7 (100)	-	1.000*			-
CES1P1	rs7187684	CC	104	6 (5.80)	98 (94.20)	-	0.225*	-	-	-
		CT	53	0 (0.00)	53 (100)					
		TT	4	0 (0.00)	4 (100)					
		C	157	6 (3.80)	151 (96.20)	-	1.000*	-	-	-
		T	57	0 (0.00)	57 (100)	-	0.090*	-	-	-
	rs11861118	AA	112	6 (5.40)	106 (94.60)	-	0.240*	-	-	-
		AG	47	0 (0.00)	47 (100)					
		GG	2	0 (0.00)	2 (100)					
		A	159	6 (3.80)	153 (96.20)	-	1.000*	-	-	-
		G	49	0 (0.00)	49 (100)	-	0.178*	-	-	-
CES2	rs11075646	CC	131	5 (3.80)	126 (96.20)	-	1.000*	-	-	-
		CG	27	1 (3.70)	26 (96.30)					
		GG	3	0 (0.00)	3 (100)					
		C	158	6 (3.80)	152 (96.20)	-	1.000*	-	-	-
		G	30	1 (3.30)	29 (96.70)	-	1.000*	-	-	-
CDA	rs602950	AA	72	3 (4.20)	69 (95.80)	-	1.000*	-	-	-
		AG	66	2 (3.00)	64 (97.00)					
		GG	23	1 (4.30)	22 (95.70)					

		A	138	5 (3.60)	133 (96.40)	-	1.000*	-	-	-
		G	89	3 (3.40)	86 (96.60)	-	1.000*	-	-	-
	rs1048977	CC	84	5 (6.00)	79 (94.00)	-	0.390*	-	-	-
		CT	59	1 (1.70)	58 (98.30)					
		TT	18	0 (0.00)	18 (100)					
		C	143	6 (4.20)	137 (95.80)	-	1.000*	-	-	-
		T	77	1 (1.30)	76 (98.70)	-	0.212*	-	-	-
TYMP	rs11479	TT	2	1 (50.00)	1 (50.00)	-	0.056*	CC	34.00	1.20 - 976.66
		CT	19	1 (5.30)	18 (94.70)				1.88	0.09 - 13.67
		CC	140	4 (2.90)	136 (97.10)				1.00	-
		T	21	2 (9.50)	19 (90.50)	-	0.176*	-	-	-
		C	159	5 (3.10)	154 (96.90)	-	0.073*	C	30.80	1.11 - 861.34

n: number of patients; OR: odds ratio; Ref. cat.: reference category; SNP: Single nucleotide polymorphism; 95% CI: 95% confidence interval.
a Pearson's Chi-square test
* Fisher's exact test

Table S19. Association of SNPs in capecitabine bioactivation pathway with treatment suspension.

Gene	SNP	Genotype	n	Treatment suspension Yes n (%)	No n (%)	χ^2	p-value [a]	Ref. cat.	OR	95% CI
CES1	rs2244613	GG	4	2 (50.00)	2 (50.00)	-	0.455*	-	-	-
		GT	48	14 (29.20)	34 (70.80)					
		TT	109	28 (25.70)	81 (74.30)					
		G	52	16 (30.80)	36 (69.20)	0.457	0.498	-	-	-
		T	157	42 (26.80)	115 (73.20)	-	0.300*	-	-	-
	rs71647871	GG	154	40 (26.00)	114 (74.00)	-	0.089*	-	-	-
		AG	7	4 (57.10)	3 (42.90)					
		AA	0	0 (0.00)	0 (0.00)					
		G	-	-	-	-	-	-	-	-
		A	7	4 (57.10)	3 (42.90)	-	0.089*	GG	3.80	0.80 - 19.99
CES1P1	rs7187684	CC	104	23 (22.10)	81 (77.90)	-	**0.024***	CC	1.00	-
		CT	53	18 (34.00)	35 (66.00)				1.81	0.86 - 3.77
		TT	4	3 (75.00)	1 (25.00)				**10.56**	**1.28 - 219.19**
		C	157	41 (26.10)	116 (73.90)	-	0.062*	C	8.48	1.05 - 174.23
		T	57	21 (36.80)	36 (63.20)	4.020	**0.044**	CC	2.05	1.01 - 4.19
	rs11861118	AA	112	26 (23.20)	86 (76.80)	-	0.162*	-	-	-
		AG	47	17 (36.20)	30 (63.80)					
		GG	2	1 (50.00)	1 (50.00)					
		A	159	43 (27.00)	116 (73.00)	-	0.473*	-	-	-
		G	49	18 (36.70)	31 (63.30)	3.137	0.076	AA	1.92	0.92 - 3.97
CES2	rs11075646	CC	131	38 (29.00)	93 (71.00)	-	0.464*	-	-	-
		CG	27	5 (18.50)	22 (81.50)					
		GG	3	1 (33.30)	2 (66.70)					
		C	158	43 (27.20)	115 (72.80)	-	1.000*	-	-	-
		G	30	6 (20.00)	24 (80.00)	0.997	0.318	-	-	-
CDA	rs602950	AA	72	18 (25.00)	54 (75.00)	1.228	0.541	-	-	-
		AG	66	21 (31.80)	45 (68.20)					
		GG	23	5 (21.70)	18 (78.30)					
		A	138	39 (28.30)	99 (71.70)	0.422	0.515	-	-	-
		G	89	26 (29.20)	63 (70.80)	0.355	0.550	-	-	-
	rs1048977	CC	84	29 (34.50)	55 (65.50)	-	0.105*	-	-	-
		CT	59	11 (18.60)	48 (81.40)					
		TT	18	4 (22.20)	14 (77.80)					
		C	143	40 (28.00)	103 (72.00)	-	0.781*	-	-	-
		T	77	15 (19.50)	62 (80.50)	4.577	**0.032**	T	2.17	1.07 - 4.57
TYMP	rs11479	TT	2	1 (50.00)	1 (50.00)	-	0.277*	-	-	-
		CT	19	3 (15.80)	16 (84.20)					
		CC	140	40 (28.60)	100 (71.40)					
		T	21	4 (19.00)	17 (81.00)	0.833	0.361	-	-	-
		C	159	43 (27.00)	116 (73.00)	-	0.473*	-	-	-

n: number of patients; OR: odds ratio; Ref. cat.: reference category; SNP: Single nucleotide polymorphism; 95% CI: 95% confidence interval.
a Pearson's Chi-square test
* Fisher's exact test

Table S20. Haplotypes of SNPs in gene *CDA* located in chromosome 1 association with overall toxicity (n = 161).

H	*CDA rs532545*	*CDA rs602950*	*CDA rs2072671*	**Frequencies**	**OR (CI95%)**	**p-value****
1	C	T	A	0.6085	1.00	---
2	T	C	C	0.3352	0.98 (0.61 - 1.57)	0.93
3	C	T	C	0.0251	3.47 (0.66 - 18.19)	0.14
4	T	T	A	0.0186	0.36 (0.05 - 2.86)	0.34
rare	*	*	*	0.0127	0.40 (0.04 - 4.02)	0.44
	Global haplotype association p-value: 0.33					

Gray: most common haplotype (Reference)
*Rare haplotype: defined as haplotype with a frequency < 0.01, no symbol is designated for this group. CI95%, 95% Confidence interval. H: haplotype, OR: odds ratio
**Adjusted by tumor localization

Table S21. Haplotypes of SNPs in gene *CDA* located in chromosome 1 association with diarrhea (n = 161).

H	*CDA rs532545*	*CDA rs602950*	*CDA rs2072671*	**Frequencies**	**OR (CI95%)**	**p-value****
1	C	T	A	0.6085	1.00	---
2	T	C	C	0.3352	1.68 (0.68 - 4.11)	0.26
3	C	T	C	0.0251	5.11 (0.40 - 65.24)	0.21
4	T	T	A	0.0186	0.00 (-Inf - Inf)	1
rare	*	*	*	0.0127	0.00 (-Inf - Inf)	1
	Global haplotype association p-value: 0.50					

Gray: most common haplotype (Reference)
*Rare haplotype: defined as haplotype with a frequency < 0.01, no symbol is designated for this group. CI95%, 95% Confidence interval. H: haplotype, OR: odds ratio
**Adjusted by drinking habit, type of capecitabine-based adjuvant treatment and histology

Table S22. Haplotypes of SNPs in gene *CDA* located in chromosome 1 association with abdominal pain (n = 161).

**H**	*CDA rs532545*	*CDA rs602950*	*CDA rs2072671*	**Frequencies**	**OR (CI95%)**	**p-value**
1	C	T	A	0.6085	1.00	---
2	T	C	C	0.3352	1.11 (0.33 - 3.76)	0.87
3	C	T	C	0.0251	0.00 (-Inf - Inf)	1
4	T	T	A	0.0186	3.37 (0.47 - 24.21)	0.23
rare	*	*	*	0.0126	0.00 (-Inf - Inf)	1
	Global haplotype association p-value: 0.73					

Gray: most common haplotype (Reference)
*Rare haplotype: defined as haplotype with a frequency < 0.01, no symbol is designated for this group. CI95%, 95% Confidence interval. H: haplotype, OR: odds ratio

Table S23. Haplotypes of SNPs in gene *CDA* located in chromosome 1 association with nausea (n = 161).

**H**	*CDA rs532545*	*CDA rs602950*	*CDA rs2072671*	**Frequencies**	**OR (CI95%)**	**p-value**
1	C	T	A	0.6085	1.00	---
2	T	C	C	0.3352	1.11 (0.33 - 3.76)	0.87
3	C	T	C	0.0251	0.00 (-Inf - Inf)	1
4	T	T	A	0.0186	3.37 (0.47 - 24.21)	0.23
rare	*	*	*	0.0126	0.00 (-Inf - Inf)	1
	Global haplotype association p-value: 0.73					

Gray: most common haplotype (Reference)
*Rare haplotype: defined as haplotype with a frequency < 0.01, no symbol is designated for this group. CI95%, 95% Confidence interval. H: haplotype, OR: odds ratio

Table S24. Haplotypes of SNPs in gene *CDA* located in chromosome 1 association with HFS (n = 161).

H	*CDA rs532545*	*CDA rs602950*	*CDA rs2072671*	Frequencies	OR (CI95%)	p-value
1	C	T	A	0.6085	1.00	---
2	T	C	C	0.3352	0.30 (0.07 - 1.37)	0.12
3	C	T	C	0.0251	0.00 (-Inf - Inf)	1
4	T	T	A	0.0186	0.00 (-Inf - Inf)	1
rare	*	*	*	0.0126	0.00 (-Inf - Inf)	1
	Global haplotype association p-value: 0.31					

Gray: most common haplotype (Reference)
*Rare haplotype: defined as haplotype with a frequency < 0.01, no symbol is designated for this group. CI95%: 95% Confidence interval. H: haplotype, HFS: hand foot syndrome; OR: odds ratio

Table S25. Haplotypes of SNPs in gene *CDA* located in chromosome 1 association with treatment suspension (n = 161).

H	*CDA rs532545*	*CDA rs602950*	*CDA rs2072671*	Frequencies	OR (CI95%)	p-value**
1	C	T	A	0.6085	1.00	---
2	T	C	C	0.3352	1.31 (0.75 - 2.28)	0.34
3	C	T	C	0.0251	1.06 (0.22 - 5.13)	0.95
4	T	T	A	0.0186	0.65 (0.08 - 5.36)	0.69
rare	*	*	*	0.0126	0.00 (-Inf - Inf)	1
	Global haplotype association p-value: 0.36					

Gray: most common haplotype (Reference)
*Rare haplotype: defined as an haplotype with a frequency < 0.01, no symbol is designated for this group. CI95%, 95% Confidence interval. H: haplotype, OR: odds ratio
**Adjusted by stage, type of capecitabine-based adjuvant treatment and PS.ECOG

Table S26. Haplotypes of SNPs in gene *CES1P1* located in chromosome 16 association with overall toxicity (n = 161).

H	*CES1P1* rs7187684	*CES1P1* rs11861118	Frequencies	OR (CI95%)	p-value**
1	C	A	0.8106	1.00	---
2	T	G	0.1584	0.90 (0.47 - 1.73)	0.76
3	T	A	0.0311	1.03 (0.31 - 3.41)	0.96
	Global haplotype association p-value: 0.95				

Gray: most common haplotype (Reference)
CI95%, 95% Confidence interval. H: haplotype, OR: odds ratio
**Adjusted by tumor localization

Table S27. Haplotypes of SNPs in gene *CES1P1* located in chromosome 16 association with diarrhea (n = 161).

H	*CES1P1* rs7187684	*CES1P1* rs11861118	Frequencies	OR (CI95%)	p-value**
1	C	A	0.8106	1.00	---
2	T	G	0.1584	0.28 (0.06 - 1.33)	0.11
3	T	A	0.0311	0.00 (-Inf - Inf)	1
	Global haplotype association p-value: 0.12				

Gray: most common haplotype (Reference)
CI95%, 95% Confidence interval. H: haplotype, OR: odds ratio
**Adjusted by drinking habit, type of capecitabine-based adjuvant treatment and histology

Table S28. Haplotypes of SNPs in gene *CES1P1* located in chromosome 16 association with abdominal pain (n = 161).

H	*CES1P1*	*CES1P1*	Frequencies	OR (CI95%)	p-value

	rs7187684	rs11861118			
1	C	A	0.8106	1.00	---
2	T	G	0.1584	2.21 (0.50 - 9.85)	0.3
3	T	A	0.0311	3.00 (0.42 - 21.49)	0.28
	Global haplotype association p-value: 0.43				

Gray: most common haplotype (Reference)
CI95%, 95% Confidence interval. H: haplotype, OR: odds ratio

Table S29. Haplotypes of SNPs in gene *CES1P1* located in chromosome 16 association with nausea (n = 161).

H	*CES1P1* rs7187684	*CES1P1* rs11861118	Frequencies	OR (CI95%)	p-value
1	C	A	0.8106	1.00	---
2	T	G	0.1584	0.00 (-Inf - Inf)	1
3	T	A	0.0311	0.00 (-Inf - Inf)	1
	Global haplotype association p-value: 0.068				

Gray: most common haplotype (Reference)
CI95%, 95% Confidence interval. H: haplotype, OR: odds ratio

Table S30. Haplotypes of SNPs in gene *CES1P1* located in chromosome 16 association with HFS (n = 161).

H	*CES1P1* rs7187684	*CES1P1* rs11861118	Frequencies	OR (CI95%)	p-value
1	C	A	0.8106	1.00	---
2	T	G	0.1584	0.39 (0.05 - 3.24)	0.39
3	T	A	0.0311	1.91 (0.28 - 13.16)	0.51
	Global haplotype association p-value: 0.49				

Gray: most common haplotype (Reference)
CI95%, 95% Confidence interval. H: haplotype, HFS: hand-foot syndrome. OR: odds ratio
